# Interactions between visceral afferent signaling and stimulus processing

**DOI:** 10.3389/fnins.2015.00286

**Published:** 2015-08-27

**Authors:** Hugo D. Critchley, Sarah N. Garfinkel

**Affiliations:** ^1^Division of Medicine, Psychiatry, Brighton and Sussex Medical School, University of SussexBrighton, UK; ^2^Sackler Centre for Consciousness Science, University of SussexBrighton, UK; ^3^Sussex Partnership NHS Foundation TrustHove, UK

**Keywords:** anxiety, arousal, autonomic, baroreceptor, cardiac cycle, emotion, neuroimaging, interoception

## Abstract

Visceral afferent signals to the brain influence thoughts, feelings and behavior. Here we highlight the findings of a set of empirical investigations in humans concerning body-mind interaction that focus on how feedback from states of autonomic arousal shapes cognition and emotion. There is a longstanding debate regarding the contribution of the body to mental processes. Recent theoretical models broadly acknowledge the role of (autonomically-mediated) physiological arousal to emotional, social and motivational behaviors, yet the underlying mechanisms are only partially characterized. Neuroimaging is overcoming this shortfall; first, by demonstrating correlations between autonomic change and discrete patterns of evoked, and task-independent, neural activity; second, by mapping the central consequences of clinical perturbations in autonomic response and; third, by probing how dynamic fluctuations in peripheral autonomic state are integrated with perceptual, cognitive and emotional processes. Building on the notion that an important source of the brain's representation of physiological arousal is derived from afferent information from arterial baroreceptors, we have exploited the phasic nature of these signals to show their differential contribution to the processing of emotionally-salient stimuli. This recent work highlights the facilitation at neural and behavioral levels of fear and threat processing that contrasts with the more established observations of the inhibition of central pain processing during baroreceptors activation. The implications of this body-brain-mind axis are discussed.

## Overview

Neuroimaging, notably using nuclear magnetic resonance imaging, has transformed human neuroscience over the last 20 years. Functional neuroimaging enables non-invasive *in vivo* evaluation of brain regions and networks supporting perceptions thoughts, feelings and continues to provide profound insight into normative brain mechanisms and functions. Neuroimaging findings have great translational potential, but so far clinical imaging biomarkers are largely limited to neurodegenerative conditions. Within autonomic neuroscience, the full impact of neuroimaging has yet to be realized. Arguably, the field has been restrained by technical challenges, for example in combining functional magnetic resonance imaging (fMRI) with detailed autonomic recording or associated experimental manipulations. However, such difficulties can and have been overcome (Gray et al., 2009a). Perhaps as relevant is a historical cultural stance that has rather underplayed the integration, across the neuraxis, of dynamic autonomic control and its contribution to perceptual, cognitive, motivational and volitional processes. This stance is increasingly challenged by neuroscientific findings and the pragmatics of therapeutic interventions. Here we review an area of autonomic neuroscience that combines human behavioral studies and neuroimaging to characterize the interaction between visceral physiology, perception and affect.

## Historical background

The contribution of bodily arousal to thoughts and feelings has been debated for many centuries. The classical Greek physician Hippocrates and his followers, seemingly arguing against an existing doctrine, proposed that the

“..*source of our pleasure, merriment, laughter and amusement, as of our grief, pain, anxiety and tears, is none other than the brain….(it) enables us to think, see and hear, and to distinguish the ugly and the beautiful, the bad and the good, pleasant and unpleasant…diaphragm nor heart.. neither take part in mental operations” (Hippocrates 400 BCE)*^1^

Relatively soon afterwards a distinct, extreme alternative is attributed to Aristotle:

“..*the brain is not responsible for any of the sensations.. the correct view [is] that the seat and source of sensation is the region of the heart….the motions of pleasure and pain, and generally all sensation plainly have their source in the heart..” (Aristotle 350 BCE)*^2^

Despite Aristotelian dominance over Western European (church-led) thought, the brain re-emerged as in the distinction between “hot” passion and “cold” reason. With Descartes, the body (including the brain) was further distanced from cognitions, and seemingly it was not until Darwin systematically highlighted the commonalities across species in the physical, physiological and behavioral expression of emotions that the link between bodily states and emotional feelings was established once again in psychological thinking (Darwin, 1872). At the turn of the nineteenth century, William James and Carl Lange both argued, with some differences, that emotional feeling states originated in physiological responses in the body:

“..*that the bodily changes follow directly the perception of the exciting fact, and that our feeling of the same changes as they occur is the emotion.”* (James, 1884)

Lange in particular attributed both positive and negative emotions to visceral vasomotor reactions (Lange, 1885/1912). Over the course of the twentieth century, there followed a series of evaluations and critiques of physiological accounts of emotion, including, for example, quantifying emotional effects of parentral adrenaline administration, from Maranon and Cantril and Hunt, to Schacter and Singer (Maranon, 1924; Cantril and Hunt, 1932; Schachter and Singer, 1962). Schachter and Singer's two stage model of emotion, followed Walter Cannon's dismissal of the contribution of peripheral physiology to emotional experience as epiphenomenological (Cannon, 1927, 1931). Schachter and Singer's model represented a compromise that acknowledged a primary, yet non-specific contribution of physiological arousal to emotions, shaped into and labeled as particular emotion types by cognitive and social expectations (Schachter and Singer, 1962). Over subsequent years, physiology has featured in a number of major models of emotion, such as labeled line (which incorporate dedicated function-specific processing architecture) (e.g., Ekman et al., 1983; LeDoux, 2000) and constructivist models of emotion (which argue that emotions are not modular phenomena, but are instead constructed from psychological influences) (e.g., Damasio, 1999; Barrett, 2006).

**Figure 1:**
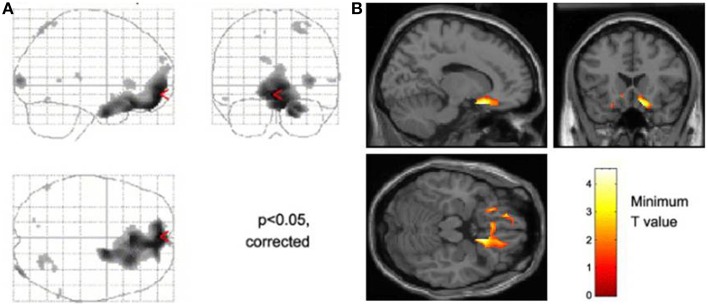
**Neural activity reflecting reducing tonic level of sympathetic electrodermal arousal**. The figure panels illustrate data presented from Nagai et al. (2004). **(A)** Decreases in skin conductance level were associated with increased activity in the ventromedial prefrontal cortex and orbitofrontal cortex this effect was independent of task in that they occurred irrespective of whether participants performed a biofeedback arousal or a biofeedback relaxation task. **(B)** Across all participants, activity within the subgenual cingulate region was associated with decreases in tonic skin conductance level.

Our own laboratory has attempted to combine cognitive neuroscience with clinical autonomic research, initially to test ideas put forward by Damasio and colleagues (Damasio et al., 1991; Bechara et al., 1997; Bechara and Damasio, 2005) concerning the influence of peripheral physiological states on decisions, thoughts and feelings. These studies combined functional neuroimaging (first using positron emission tomography, PET, then magnetic resonance imaging) with monitoring of autonomic responses evoked by performance of cognitive emotional or effortful tasks (Critchley et al., 2000a,b, 2005a,b). Across experiments, a characteristic pattern of activity was associated with states of psycho-physiological arousal, generally irrespective of whether the participants were processing salient emotional information or performing demanding cognitive or effortful motor tasks. Such challenges commonly evoke an enhancement of activity within dorsal anterior cingulate cortex (extending caudally to mid cingulate) accompanied by bilateral mid to anterior insular cortex activity. Activity in these areas typically correlate with autonomic change; shifts in sympathovagal balance (toward sympathetic arousal and parasympathetic withdrawal) whether measured by electrodermal activity, changes in pupil size, heart rate acceleration or heart rate variability (Critchley et al., 2002, 2003, 2005a,b). This coupling of motivational behavior with adaptive changes in bodily-state provides an integrative account of why certain regions process both mental and physiological information. For example, regions such as “cognitive” anterior cingulate activate when both mental and physiological resources are diverted to meet behavioral challenges. Observed attenuation of sympathetic arousal in patients with lesions affecting dorsal anterior/mid cingulate (Tranel, 2000) during motor and cognitive effort further supports the notion that rostromedial cortex hosts a visceromotor center that drives action-ready autonomic states during psychological arousal. Interestingly, the ventromedial prefrontal cortex and subgenual cingulate region appears, across a number of experiments, to be “antisympathetic” and/or parasympathetic: For example, the tonic level of sympathetic electrodermal arousal is negatively correlated with activity within this region (Nagai et al., 2004) (Figure 1), and correspondingly, correlations between high frequency heart rate variability (an index of parasympathetic cardiac control) and ventromedial prefrontal cortical activity are also observed. In an elegant illustration, Wager and colleagues showed that increases in heart rate induced by the stress of “social evaluation” are independently predicted by increased activity within dorsal anterior cingulate and decreased activity within ventromedial prefrontal cortex (Wager et al., 2009). In contrast to anterior cingulate cortex, across different studies evidence points to a role of insular cortex in representing (mapping) states of autonomic arousal and visceral change (Critchley et al., 2004; Pollatos et al., 2007).

A core aspect to stress-induced physiological arousal is the suppression of the normal baroreflex response, to allow heart rate and blood pressure to rise together. During experimental induction of psychological stress, activity within dorsal cingulate, bilateral insula, amygdala and dorsal brainstem (midbrain periaqueductal gray matter) predicted the magnitude of beat-by-beat baroreflex suppression (Gianaros et al., 2012). It is noteworthy that each of these regions is implicated, indicating that dynamic interaction between afferent and efferent axes of autonomic control extend beyond brainstem to forebrain (both subcortical and cortical) centers. The contribution of afferent autonomic feedback to the expression of physiological arousal in emotion is consistent with the observed neural correlates of mental stress. Both insular cortex and amygdala are sensitive to the presence of threat-induced autonomic responses: In a fear conditioning study that compared patients with autonomic failure to healthy controls, the absence of an autonomic response to threat stimuli was associated with attenuated activity within amygdala and mid insula. Insular cortical responses, in both mid and right anterior regions, were observed to reflect the further integration of physiological feedback with the conscious processing of threat stimuli, achieved experimentally by comparing responses to supraliminal and subliminal conditioned threat stimuli between autonomic failure and control participants (Critchley et al., 2002). While the amygdala is a key region thought to reflect fear/threat, emotion, and general salience (Santos et al., 2011), its apparent “activation” when monitored using fMRI can be subject to potential confounds derived from a venous origin (Boubela et al., 2015). Moreover, the experience of fear can be experienced in the absence of amygdala activation, as demonstrated via inhalation of 35% CO_2_ in patients with bilateral amygdala damage (Feinstein et al., 2013). Together these studies highlight a need to review past amygdala lesion and activation studies with respect to interpreting it as an obligatory substrate for emotions.

The general observation that, in health, subliminal threat stimuli can evoke physiological arousal responses provides support for the automatic primacy of bodily responses to emotional experience. Such evidence is very relevant to peripheral theories of emotion, where the feedback of bodily response is proposed to be the basis for emotional feelings (Lange and James, 1967), in contrast to a common process generating feelings and physiological change. The information fed back from the automatic bodily response can then guide perceptions and decisions as “somatic markers” (Damasio et al., 1991; Bechara et al., 1997; Bechara and Damasio, 2005). Within this theme, Katkin and colleagues conducted a study where participants were subliminally presented (using backward masking) two stimuli, one of which predicted the occurrence of a later shock. At each trial, during a delay following the stimulus presentation, the participant was asked to judge whether they thought they would receive a shock. Without conscious awareness to enable discrimination between the stimuli, participants should have, in theory, performed at chance on this “trace conditioning” task. However, it was observed that some individuals, preselected as being “interoceptively aware” based on their accurate performance on a heartbeat detection task, were able to estimate well above chance whether a subliminal stimulus was paired with a later shock (Katkin et al., 2001). The interpretation was that those individuals most sensitive to their bodily responses, i.e., able to access the arousal responses generated automatically by the subliminal threat, could effectively use that information in their decision-making.

## Interoceptive ability

**Figure 2:**
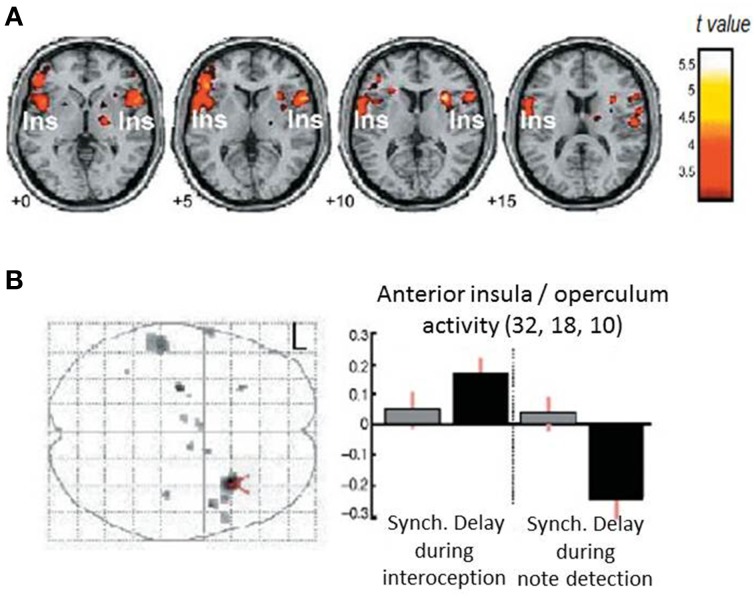
**Neural correlates for interoceptive processing**. The figure panels illustrate data presented in Critchley et al. (2004). **(A)** Activity in the insula is enhanced during interoceptive attention relative to an exteroceptive control condition (i.e., trials where attention is directed to the heart vs. trials where attention is directed to the notes alone). **(B)** Neural activity reflecting the interaction between interoceptive/exteroceptive attention (heart vs. notes) and feedback delay (tones synchronous or delayed with respect to the heartbeat). For delayed stimuli, activity in right insula is enhanced during interoceptive focus and reduced during exteroceptive focus.

The measurement of individual differences in interoceptive sensitivity/accuracy has over the years gravitated toward heartbeat detections tasks. Correlations with other axes of interoception (e.g., gastric filling) have reassured people that the ability to perceive individual heartbeats at rest can lead to inferences about an individual's more general sensitivity to internal bodily responses and arguably, by extension, their impact on emotional processes. Heartbeat detection tasks include the “Schandry task”: counting heartbeats at rest over a fixed interval (comparing reported to actual number of heartbeats measured using electrocardiography) (Schandry, 1981) and the Whitehead/Katkin task where people judge the timing (synchronous or delayed) of external auditory or visual stimuli relative to the heart beats that triggered the stimuli (Whitehead et al., 1977; Katkin et al., 1983; Wiens and Palmer, 2001). Neuroimaging studies (Critchley et al., 2004; Pollatos et al., 2007) associate performance on heartbeat detection tasks with engagement of right insular cortex in particular as part of a wider network of regions including anterior cingulate cortex (Medford and Critchley, 2010). These studies confirm a role for insula engagement in interoceptive processing, providing further insight into how emotional feeling states especially of anxiety, are supported within interoceptive representations (Critchley et al., 2004; Paulus and Stein, 2006). Interestingly, the response of part of right anterior insula differed during a Whitehead/Katkin task, both according to whether participants' attention is focused “interoceptively” on their heartbeats or exteroceptively only on the quality of the auditory stimuli, and according to the timing of the external stimulus (presence or absence of a delay relative to heartbeat) (Critchley et al., 2004) (see Figure 2). This interaction suggests, at least for right anterior insular cortex, that there is a fine-grained integration of external sensory information with representation of individual heartbeats. Anterior insular cortex therefore responded to mismatched timing between internal and external signals.

## Insula and detection of interoceptive mismatch

Peripheral theories of emotion superficially appear to predict that interoceptive sensitivity would be associated with enhanced emotional responsivity and correspondingly increased vulnerability to emotional disorders, including clinical anxiety. While “good heartbeat detectors” may be over represented among anxiety patients, interceptive accuracy does not account sufficiently. Attention to, appraisal and expectation of physiological arousal states interact with awareness to fuel symptoms such as anxiety (Clark, 1986; Wild et al., 2008; Paulus and Stein, 2010; Garfinkel et al., 2015). Contrasting the effects of false and true physiological (heartbeat) feedback during an MRI study, Gray and colleagues (Gray et al., 2007) showed that processing and subjective rating of neutral faces was enhanced in the presence of false physiological arousal, consistent with the notion that “unattributed arousal” was assigned to an ambiguous cause: the presence of neutral facial expressions. Correspondingly, intrinsically arousing emotional faces did not show the same enhancement effect. This behavioral effect supports the idea that unexpected physiological arousal contributes to states of anxiety or feelings of threat by donating salience to coincidental potential causes. In this false feedback study, neuroimaging identified right anterior insular cortex and amygdala activity as mediating processing of interoceptive/exteroceptive error evoked by the false feedback to predict the enhancement of perceived intensity of neutral faces.

These data point to the coupling of emotional states autonomic activity and feedback via insular regions. The processing of threat is attenuated in different in people with absent autonomic responses who show less engagement of insula and amygdala in response to threatening stimuli, moreover mid and anterior region of right insula link the representation of autonomic arousal to conscious awareness of the likely cause of the autonomic arousal in a manner consistent with the two stage model of emotion proposed by Schachter and Singer (1962), wherein emotions are constructed from the interpretation of physiological change in the concurrent cognitive context. Individual differences in interoceptive accuracy (heartbeat detection) influences emotional reactivity, and again the insular cortex is the dominant substrate for accessible interoceptive representation (Critchley et al., 2004). A major role of insula in processing physiological change lies in the processing of the mismatch between perceived and inferred/expected bodily state in an attention- dependent manner. Here unattributed arousal can alter emotional appraisal of face stimuli (via insula and amygdala) in a manner that appears to enhance the salience and potentially threatening nature of “ambiguous” neutral stimuli (Gray et al., 2007).

## Baroreceptor signaling and cardiac timing

In discussing the contributions of bodily physiology to the processing of stimuli and the generation emotional feelings, there appears at least in our view to be a primacy or dominance of feedback of cardiovascular influences. While some autonomic indices such as electrodermal responses or pupil size may be sensitive indicators of emotional/affective reactions, states of cardiovascular arousal, particularly strong fast heartbeats, are felt as potent influences on subjective emotion. What is noteworthy about the feedback from the heart and great vessels is that the signal is phasic, pulsing with each heartbeat. The cardiac cycle describes the phases of atrial and ventricular filling and discharge of blood from the heart into the wider circulation. With each systole, blood leaves the left ventricle into the aorta and carotids where stretch receptors in the vessel walls (baroreceptors) are activated. These signals are conveyed centrally to the brainstem via the vagus and glossopharyngeal cranial nerves. Within the medulla, these phasic signals are processed to inform the baroreflex, whereby blood pressure is controlled by the slowing of the heart (parasympathetic vagus) after strong heart beats and inhibition of muscle sympathetic nerve activity to attenuate vasoconstriction of vascular beds. Strong baroreceptor discharges are responded to by adaptive adjustments to maintain perfusion pressure. The same channel of afferent information flowing from aortic/carotid sinus baroreceptors to the brain is presumed to be the basis of heartbeat detection and the feeling states of physiological arousal accompanying motor and emotional behaviors. We can explore experimentally the effects of this viscerosensory pathway on other perceptual and mental processes without necessarily changing the overall state of cardiovascular arousal, since baroreceptor signals occur in bursts and are quiet between heartbeats: A brief stimulus presented at systole is processed concurrently with aortic/carotid baroreceptors signaling, while this is not the case for a stimulus presented at diastole (Lacey and Lacey, 1970; Rau and Elbert, 2001).

## Heartbeat timing experiments

There is an established body of literature that has pursued this type of experiment, and associated theory. Broadly, the majority of these studies indicate that stimuli presented concurrently with baroreceptor activation (natural activation at systole) or augmented with external neck suction) appear to be inhibited. This is particularly the case for painful stimuli (e.g., brief electrocutaneous shock) where there is attenuation of pain evoked potentials, nociceptive motor and autonomic reflexes and the perception of pain presented at the time of baroreceptor activations relative to quiescent periods (Dworkin et al., 1994; Rau and Elbert, 2001; Edwards et al., 2002; McIntyre et al., 2006, 2008). Baroreceptor stimulation is reported to engender similar blunting on stimulus processing. The observations of Lacey and Lacey (Lacey and Lacey, 1970, 1978) with respect to these and related cardiac cycle effects (including cardiac deceleration in anticipation and orientation, and acceleration for action and response) led to the formulation of a general principle that heartbeat/baroreceptor stimulation is generally inhibitory, perhaps representing a distracting stream of information when one needs to survey one's surroundings, but which can help facilitate motor behaviors, including fight and flight responses, in part by devaluing external distraction.

The conjunction of arterial baroreceptor activation and external sensory stimulation has revealed some interesting autonomic effects. For example, contrary to the notion that sympathetic activation is a general indicator of psychophysiological arousal, the sympathetic responses to an arousing electrocutaneous shock at systole are distinct between electrodermal measures and muscle sympathetic nerve activity: the shock evokes a sympathetic skin response (irrespective of timing in relation to baroreceptor activation) but causes inhibition of the burst-firing of muscle sympathetic nerve bundles (Donadio et al., 2002; Wallin, 2007). The predicted consequence is therefore a transient drop in blood pressure. This inhibition of muscle sympathetic nerves diminishes through habituation if the stimulus is repeated over ensuing systoles. Most interestingly, there is much less habituation in blood-phobic fainters (Donadio et al., 2007), suggesting a physiological signature underlying the propensity to faint, wherein the interaction of interoceptive signals from the heart with the processing of directly threatening stimuli is linked to a stereotyped emotional behavioral reaction.

## Neural correlates of heartbeat timing effects on sensory processing

**Figure 3:**
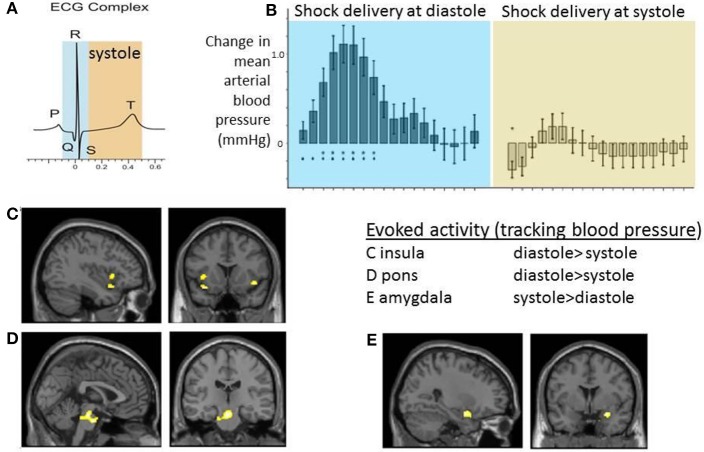
**Neural activity reflecting interaction between cardiac afferent information within cardiac cycle and electrocutaneous shock processing**. The figure panels illustrate data presented in Gray et al. (2009b). Electrocutaneous shocks administered at cardiac systole (around T wave of electrocardiogram; ECG) inhibit normal blood pressure response and decrease blood pressure-related activity with insula and pons while increasing activity in amygdala (relative to diastole). **(A)** Timing of electrocutaneous stimuli relative to ECG R-wave: aiming to trigger at systole around ECG T wave, and to trigger at diastole (immediate presystole period). In fact for practical purposes stimulus events were triggered in a predictive way from pulse oximetry data and the accuracy of relationship to concurrently recording ECG confirmed *post-hoc*. **(B)** Group data illustrating the differential effect of shock timing in cardiac cycle on beat-to-beat mean arterial blood pressure responses across the group. Systole was observed to attenuate blood pressure increase to shock. **(C–E)** Group BOLD activity tracking blood pressure following shock delivery was contrasted for events at systole vs. diastole. Activity within C. bilateral insular cortex regions and D. dorsal brainstem (mid pons) was greater at diastole compared to systole. E. Group activity in right amygdala was greater at systole compared to diastole.

Certainly such observations indicate the modulatory influence of signals from the heart and great vessels on external stimulus processing. Moreover, the habituation effect and link to blood phobia suggest that these mechanisms involved supratentorial regions supporting expectation, attentional and emotion. A neuroimaging study exploring the brain subregions supporting interactions between electrocutaneous shock and baroreceptor activation/heart timing combined a number of physiological indices (including non-invasive beat-to-beat blood pressure) with event-related functional magnetic resonance imaging (Gray et al., 2009b). Compared to timing at diastole, electrocutaneous shocks delivered at systole were associated with a flattening of blood pressure response which was coupled to changes in the activity of mid pons, bilateral anterior insula, and right amygdala. Interestingly, while this activity within pons and insula was attenuated to stimuli presented at systole, the effect in amygdala went in the opposite direction: blood-pressure coupled activity in amygdala was greatest at systole (Figure 3). There was further evidence to link these effects to baroreflex control: individual differences in high–frequency heart rate variability (an index of parasympathetic cardiac control) particularly predicted the differential evoked activation of insula at systole compared to diastole. Trial-by-trial changes in heart rate variability measure were predicted by systole/diastole activity difference within periaqueductal gray matter and amygdala (Gray et al., 2009b). This study therefore provided evidence for integration across viscerosensory, affective and autonomic systems within the brain in response to a salient physical stimulus.

Using electroencephalography, to extend earlier knowledge regarding baroreceptor influences on pain processing, Gray and colleagues modulated participants' expectation of painful shock with visual cues. Early components of pain-evoked potentials (e.g., N2) were not significantly affected by expectation or systole/diastole timing. However, the magnitude of a later component (P2, occurring around 400 ms after shock delivery and distributed across central and right lateral scalp regions) differentiated between expected and unexpected shocks presented at diastole. Baroreceptor activation (i.e., the timing of stimuli at systole) abolished the difference between expected and unexpected shock on this P2 component. This observation was consistent with a simple model that suggests this later component of pain processing is gated by attention, but furthermore the operation of this attentional gate is conditional upon a baroreceptor gate: i.e., signals from the heart and great vessels exert their influence at an attention-dependent stage that follows initial sensory mapping to brain (Gray et al., 2010). Thus, at least for pain (and there is evidence for other sensory stimuli), natural baroreceptor afferent effects on sensory processing appear to occur at a secondary stage after initial sensory representation. Of relevant interest, however, are the findings of a recent magnetoencephalographic (MEG) study, which showed that the magnitude of heartbeat evoked potentials (an index of cortical representation of signals from the heart and great vessels) did in fact predict whether or not a fine grained visual stimulus (low resolution circular grating) entered conscious awareness (Park et al., 2014). Nevertheless, this MEG study did not show a direct effect of systole/diastole timing on stimulus detection.

## Heartbeat timing effects on emotional processing

The core observation that the processing of emotive (pain) stimuli are modulated/gated by the cardiac cycle led to the question as to whether other types of salient emotional stimuli would be changed in value by these phasic cardiac afferent signals. Emotional facial expressions have been widely used in emotion research and affective neuroscience to probe and engage brains systems. This approach has accommodated both labeled line theories of specific discrete emotions (Ekman and Friesen, 1971; Ekman and Cordaro, 2011) and more dimensional constructionist views about emotional processing (Barrett, 2006). Comparing the effects of presenting brief, but nevertheless overt, pictures of different emotional faces at systole and diastole demonstrated modest differences (c.1.5%) in subsequent heart rate for disgust and happy stimuli, both showing greater slowing of heart rate to these facial expressions when processed at systole compare to diastole. No significant effects were seen for sad and neutral faces (Gray et al., 2012). Moreover for disgust expressions there was also significant change in ratings of emotional intensity (measured rather insensitively on a 5 point Likert scale). This study was conducted during simultaneous brain imaging with fMRI, which revealed an area of periaqueductal gray matter whose activity mirrored the cardiac timing effect on stimulus processing and negatively emotional predicted ratings. Specifically for disgust, there was a similar convergent effect within left mid orbitofrontal cortex (Gray et al., 2012). Thus, phasic baroreceptor signals contribute to the processing of some emotions, but it is noteworthy that this study did not examine fear stimuli. This oversight was remedied in a further set of studies, motivated by the possibility that the heart timing effect may have the potential to differentiate and help people with anxiety disorders and fear-related conditions such as phobia.

The observation of amygdala engagement in the pain study also contributed to the motivation for a study similar to that of Gray et al. (2012), but combining fMRI with the presentation of fearful and neutral facial expressions at different phases of the cardiac cycle (systole and late diastole). The presentation of fear face stimuli at systole compared to diastole evoked an enhancement of subjective perception of the emotion, with reported intensity ratings changing by around 6%. A trend in the opposite direction was noted for neutral faces, with intensity ratings enhanced slightly at diastole. This effect of heart timing on emotion perception was associated with bilateral engagement of amygdala, the magnitude of which, particularly for the right amygdala, correlated with individual differences in trait anxiety levels (Garfinkel et al., 2014).

In the same set of investigations, a further effect was noted: baroreceptor activation, (as inferred from timing stimuli at systole) enhanced the detection of threat signals (fear faces) but not of other emotions (Garfinkel et al., 2014). In a rapid serial visual presentation (RSVP, attentional blink paradigm), emotional faces presented among distracting masks (at the border of conscious awareness) are more likely to ‘break through’ to conscious awareness. This “emotional attentional blink” effect shows that salience of affect-laden stimuli grabs attention at an early stage (Anderson and Phelps, 2001), an effect that is abolished in amygdala lesioned participants (Anderson and Phelps, 2001). In a behavioral study, timing emotional faces to phases of the cardiac cycle, produced a significant facilitation of this attentional grab for fear faces presented at systole compared to diastole, but not for disgust, happy or neutral faces (though notably there was a trend for detection of neutral faces to be better at diastole). The magnitude of change was noteworthy with around a 9% average shift in detecting fear faces at systole compared to diastole. Interestingly, not all of the healthy participants of the study demonstrated this average effect: 3/19 people showed no differences between systole and diastole for fear faces detection and, 2/19 showed small effects in the opposite direction. At the other extreme, 3 people showed 25% or greater improvement in detection of fear faces at systole (Garfinkel et al., 2014). This study on fear detection clearly enriches the picture presented above regarding the impact of heart signals on early sensory processing (Gray et al., 2009b; Park et al., 2014). In summary, the cardiac timing effects markedly and selectively influence the detection and emotional appraisal for fear signals, at least for facial expressions. The direction of these effects on fear processing is the opposite of what had previously been the generally received wisdom that baroreceptor afferent inputs are inhibitory to stimulus processing, and the opposite of what is observed for the more direct challenge of brief painful (electrocutaneous) stimuli. The effects in the brain appear mediated through systems, notably the amygdala, known to be engaged in threat processing and its integration with afferent information regarding autonomic arousal.

## Conclusions and future research

The notion that states of autonomic arousal contribute to emotional processing has a long history. Its relevance is particularly noted in appraisal models of emotion and incorporated into cognitive models of panic and anxiety. Inspirational work in the 1970's and 1980's highlighted a particular role of baroreceptor activation in conveying signals to the brain about the state of cardiovascular arousal, beyond its proximate role in the reflex control of blood pressure. Heart timing experiments (reinforced by studies employing external mechanical manipulation of baroreceptors) highlighted inhibitory effects on processing of stimuli, notably the processing of pain, as expressed in motor reflexes, electrocortical responses and subjective judgments. Recent work, particularly from our own laboratory, has highlighted opposite effects on the processing of fear signals of potential threat, notable facial expressions. Here baroreceptor activation facilitates the detection of fear and augments the attribution of emotional salience. Brain imaging of these effects has helped not only define the levels of visceroaffective integration within brain, but imaging has also identified objective markers and potential targets of intervention since these same areas are typically implicated in affective psychopathology, including the expression of anxiety disorders. It is therefore a goal of future clinical research to explore and exploit these observations. Fundamentally using or manipulating afferent signals from the heart to enhance or diminish the detection and processing of affective stimuli has potential application for characterizing and stratifying patient groups for different anxiolytic treatment, and may provide a means of enhancing the interventions themselves, for example by integrating physiological signals with computerized cognitive behavioral therapies. More options may emerge as we learn more about the neurochemistry and functional architecture supporting these effects.

### Conflict of interest statement

The authors declare that the research was conducted in the absence of any commercial or financial relationships that could be construed as a potential conflict of interest.

## Footnotes

^1^Hippocrates. 400 BCE. On the Sacred Disease.

^2^Aristotle. 350 BCE. Parts of Animals.
